# Association between thermal balance of the brain, inflammation and response to therapy in patients with schizophrenia.

**DOI:** 10.1192/j.eurpsy.2023.2316

**Published:** 2023-07-19

**Authors:** V. Kaleda, S. Zozulya, T. Klyushnik, D. Tikhonov, A. Simonov, O. Shevelev, M. Petrova, E. Mengistu

**Affiliations:** 1 Department of youth psychiatry; 2 Department of neuroimmunology; 3 Laboratory of Evidence-Based Medicine and Biostatistics, Federal State Budgetary Scientific Institution Mental Health Research Center; 4 Laboratory of Clinical Neurophysiology, Federal Research and Clinical Center of Intensive Care Medicine and Rehabilitology; 5 Department of General Pathology and Pathological Physiology (named after Frolov V.A.), Medical Institute, “Peoples’ Friendship University of Russia”; 6 Deputy Director for scientific and clinical work, Federal Research and Clinical Center of Intensive Care Medicine and Rehabilitology; 7 Department of Anesthesiology and Intensive care with course of rehabilitation, Medical Institute, “Peoples’ Friendship University of Russia”; 8 Laboratory of comorbidity and autonomic dysfunction studies, Federal Research and Clinical Center of Intensive Care Medicine and Rehabilitology, Moscow, Russian Federation

## Abstract

**Introduction:**

Disruption of cerebral thermal homeostasis accompanies various CNS diseases. Presumably, (neuro)inflammation and the changes of temperature heterogeneity of the cerebral cortex may be interrelated links in the pathogenesis of schizophrenia.

**Objectives:**

to study the association between the brain thermal balance indicators, inflammatory markers and clinical features of the disease in patients with schizophrenia during therapy.

**Methods:**

37 patients aged 16 to 46 years with schizophrenia (F20, ICD-10) were examined. Clinical examination included psychometric assessment using PANSS, HDRS, and YMRS scales. Cortical temperature was determined by microwave radiometry. Temperature heterogeneity was assessed by calculating the Pearson correlation coefficient between temperature indicators in 9 symmetrical areas of the cerebral cortex. The activity of the proteolytic system of inflammation (ratio of leukocyte elastase (LE) and α1-proteinase inhibitor (α1-PI) activity) and the level of autoantibodies to S100B and MBP antigens were determined in patients’ blood.

**Results:**

Low temperature heterogeneity is related to an increase in the activity of the proteolytic system of inflammation and a good response to therapy in most patients. High temperature heterogeneity is associated with insufficient activity of the proteolytic system of inflammation and the development of autoimmune reactions, which is accompanied by a more severe course of the pathological process and, in most cases, treatment resistance.

**Conclusions:**

The association between the features of the thermal balance of the brain and inflammatory markers confirms the hypothesis of their role in the pathogenesis of schizophrenia. Temperature heterogeneity of the brain can serve as a criterion for predicting of therapeutic response in patients with schizophrenia.

**Disclosure of Interest:**

None Declared

